# The Meaning and the Treatment of Dropsy

**Published:** 1898-07-09

**Authors:** 


### THE MEANING AND THE TREATMENT OF DROPSY.

The paper read by Dr. W. Ewart at the last meeting of the Royal Medical and Chirurgical Society on the Treatment of Tubal Nephritis raises many interesting questions; and, although we may not be ready to accept all his conclusions, we think the paper a most valuable one, especially in regard to the suggestions contained in it concerning the use and the *modus operandi* of lymphatic drainage. The usual well-known features of tubal nephritis are the dropsy, the anæmia, the oliguria, and the cachexia and marasmus, the latter being often disguised by the persistent œdema. Dr. Ewart's paper was chiefly directed to a consideration of the dropsy, which he looked upon, on the one hand, as Nature's protection and remedy against fatal intoxication, and on the other as giving us an opportunity of granting physiological rest to the kidneys by setting up a fresh emunctory channel. In itself, he says, œdema is harmful when persistent by oppressing the tissues, choking the lymphatic circulation, and impeding metabolism. It hinders nutrition both locally and generally, and its usual treatment by horizontal rest in bed further depresses the vital functions. What he recommends, then, is that the œdema should be encouraged to collect in the lower limbs by slanting the bed, so that while the trunk and the upper limbs, relieved of their puffiness, can be restored to healthier action by massage, positive relief to the tissues of the entire body, to the serous cavities, when implicated, and to the lymphatic circulation may be given by acupuncture or incision and drainage. Nay, he would go further, and in some cases of tubal nephritis, in which dropsy is either absent or very small in quantity, he would produce an artificial form of dropsy by pressure applied round a lower limb, so as to be able to obtain a flow of fluid from an incision. It must be clearly recognised that the incision or the acupuncture is used not merely as a means of removing dropsy—a means, therefore, to be postponed until the dropsy itself is doing harm—but is used with the object of supplying a fresh emunctory channel, and thus relieving the kidney and giving it a physiological rest. The matter may be put in this way: The occurrence of dropsy is a protective mechanism, but itself does harm. If, however, it can be removed as it is formed, one gets the good without the harm; and there can be little doubt that Dr. Ewart is right; the benefit derived from Southey's tubes is sometimes far greater than can be explained by the mere diminution of dropsy. What we may suppose that it does is to allow the dropsy to take place innocuously. With our more perfect knowledge of aseptic methods drainage of dropsy ought in the future to be far more useful as a therapeutic method even than it has been in the past. Incidentally it is pointed out that one great advantage derived from its employment is that while the flow continues a liberal diet can at times be adopted, and that it is thus possible to restore the cardiac energy and nutrition which, in the presence of an unremoved dropsy, tends to fail. Needless to say, the paper was criticised, and especially was it pointed out that in cases of large white kidney there was an interstitial change, and that it was not a disease of the tubes alone. Perhaps, however, we do not yet know how far the permanence of the interstitial change may not depend upon the tubal mischief being more or less prolonged, and it is fair to believe that what will help the tubes to recover quickly will lessen the chances of permanent tissue changes taking place.

